# Comparative Analysis of Early Complications and Length of Hospital Stay After Laparoscopic and Open Inguinal Hernia Repair

**DOI:** 10.3390/life16050731

**Published:** 2026-04-28

**Authors:** Bartosz Socha, Luiza Sannikova, Michał Dyaczyński, Georgii Gogichev, Marcin Basiak

**Affiliations:** 1 Department of General, Minimally Invasive and Oncological Surgery, Silesian Hospital in Cieszyn, 43-400 Cieszyn, Poland; 2 Independent Researcher, 121552 Moskow, Russia; 3 Department of Internal Medicine and Clinical Pharmacology, Medical University of Silesia, 40-055 Katowice, Poland

**Keywords:** surgery in Poland, laparoscopic surgery, length of hospital stay, patient’s age, general surgery

## Abstract

Inguinal hernia repair remains one of the most frequently performed surgical procedures, but the problem of early postoperative complications remains significant, particularly in the context of surgical technique selection and patient age. The aim of this study was to compare the incidence of early complications and the length of hospital stay in patients undergoing laparoscopic and open inguinal hernia repair. To achieve this goal, a retrospective study was conducted, analyzing data from 172 patients who underwent laparoscopic and open mesh repair. The results showed that no early complications occurred in patients undergoing laparoscopic repair, whereas complications were reported in 6.4% of patients undergoing open repair, primarily in older adults. The average hospital stay was also shorter with the laparoscopic approach. These data suggest advantages of laparoscopic surgery, especially for elective procedures, which may help reduce complication rates and accelerate recovery. The practical significance of the work is that the obtained results can be used to optimize the selection of surgical technique in the treatment of inguinal hernia, especially in the context of limited financial resources and the need to improve the quality of medical care in Polish medical facilities.

## 1. Introduction

Inguinal hernia is one of the most common surgical conditions requiring surgical intervention. Epidemiological studies indicate that approximately 25% of men and up to 3% of women will need surgical treatment for this condition during their lifetime. Inguinal hernia repairs constitute a significant part of general surgery procedures. While early postoperative complications are generally uncommon, their occurrence remains clinically relevant in specific patient groups, particularly in elderly patients and those with comorbidities. Among the existing methods for inguinal hernia repair, the most widely used are the Lichtenstein method and the laparoscopic TAPP technique. The former is considered the standard for open surgery due to its simplicity, high effectiveness, and relatively low cost. The laparoscopic approach is associated with reduced surgical trauma and faster postoperative recovery, although it is also linked to a specific spectrum of complications, which may differ from those observed in open techniques. However, the choice between these approaches often depends not only on clinical indications but also on equipment availability, surgeon’s skill level, and economic factors [1,2,3].

According to a previous study, the Lichtenstein technique remains the most commonly used method among surgeons in Poland, whereas laparoscopic approaches such as TAPP and TEP are still relatively infrequently applied in routine clinical practice [4]. The limited dissemination of modern methods may be related to factors such as variability in training opportunities, differences in surgical experience, and resource availability across healthcare settings [5]. Another study also indicates a persistent tendency to choose the traditional open approach, even if there are clinical indications for minimally invasive interventions [6].

Although early postoperative complications are generally uncommon, their occurrence may vary depending on patient-related factors, including age and comorbidities, as well as the surgical approach used. In particular, elderly patients represent a growing subgroup with potentially higher perioperative risk [7,8,9,10,11,12].

Therefore, despite a significant number of publications, the issue of comparative analysis of the TAPP method and the Lichtenstein method remains open, especially when assessing treatment outcomes in patients of different age groups. In the context of the Polish healthcare system, where a disparity persists between modern international recommendations and actual clinical practice, further research in this area remains warranted, particularly in the context of differences between international recommendations and everyday clinical practice.

The aim of the study was to conduct a comparative analysis of the incidence of early postoperative complications and the duration of hospital stay in patients undergoing inguinal hernia repair using the TAPP and Lichtenstein methods, with particular emphasis on the age factor.

## 2. Materials and Methods

This study was conducted as a retrospective analysis of clinical data from patients undergoing inguinal hernia repair at a surgical ward in a multidisciplinary, district-level hospital in Poland, reflecting routine clinical practice in a non-tertiary care setting. The aim of this stage of the study was to assess the incidence of early postoperative complications and the length of hospital stay depending on the chosen surgical technique—laparoscopic (TAPP) or open (Lichtenstein method). Particular attention was paid to the influence of patient age on the incidence of complications. In this institution, patients are routinely admitted one day prior to surgery, undergo the procedure on the following day, and are discharged the next morning, which influences the overall length of hospital stay.

The study period was from January to June 2023 inclusive. A total of 172 patients who underwent inguinal hernia repair were selected. Thirty patients underwent laparoscopic TAPP surgery, while the remaining 142 underwent the Lichtenstein technique. All patients underwent elective or emergency surgery, depending on clinical indications and resource availability.

The study included patients of both sexes (men and women) aged 19 to 92 years. The main inclusion criteria were the presence of an inguinal hernia, the need for surgical intervention, the availability of complete medical records for each case (including medical history, surgical data, and postoperative follow-up), and the patient’s written informed consent to use the anonymized data for research purposes. Patients with recurrent hernias, multiple surgical procedures in the same area, and missing data on the length of hospital stay or postoperative outcomes were excluded. Patients were not randomly assigned to the surgical technique. The choice between TAPP and Lichtenstein repair was made based on routine clinical decision-making, taking into account patient-related factors (including age, comorbidities, and anesthetic risk), the urgency of the procedure, surgeon experience, and the availability of laparoscopic equipment.

The study included patients who met the following criteria:

− Diagnosis of inguinal hernia requiring surgical treatment;
− Age ≥ 18 years;
− Availability of complete medical records, including perioperative and postoperative data.

Exclusion criteria were as follows:

− Recurrent inguinal hernia;
− History of previous surgical procedures in the same anatomical region;
− Incomplete clinical data regarding postoperative outcomes or length of hospital stay.

Data collection was conducted through examination of the patients’ paper and electronic medical records. The following parameters were examined: patient age and gender, type of surgery (elective or emergency), surgical technique used, length of hospitalization, and the presence and nature of early postoperative complications. These included hematomas, wound infections, seromas, bleeding, acute urinary retention, and other complications noted within the first three days after surgery. Clinical data were collected retrospectively by two physicians and independently verified by a third investigator from the same center.

Descriptive and comparative statistics were used to analyze the collected data. Mean values for age, hospitalization duration, and incidence of complications were calculated in each study group. Statistical analysis was performed using Student’s *t*-test for independent samples (for quantitative variables) and the chi-square test or Fisher’s exact test (for qualitative variables). Results with *p* < 0.05 were considered statistically significant. Anesthetic risk was assessed using the ASA (American Society of Anesthesiologists) scale, while comorbidities were described qualitatively without the use of point scales.

Data visualization and statistical calculations were performed using Python (version 3.10) with standard scientific libraries (e.g., NumPy, Pandas, Matplotlib). The pandas library was used to organize and analyze tabular data, while the matplotlib library was used to create graphs depicting the distribution of complications and length of hospital stay. This allowed not only for the quantitative assessment of differences between groups but also for a clear presentation of the identified relationships.

The TAPP method, used in 30 patients, is a transperitoneal laparoscopic approach in which a mesh graft is implanted through the abdominal cavity and then secured in the preperitoneal space. Procedures were performed under general anesthesia using 5 and 10 mm trocars. All procedures were performed by surgeons experienced in laparoscopic operations. Particular attention was paid to the mesh application technique and ensuring reliable implant fixation to prevent recurrences and complications.

The Lichtenstein method, used in most patients, was performed using a standard open approach and implantation of a mesh endoprosthesis into the inguinal canal. Procedures were primarily performed under spinal anesthesia. In most cases, 10 × 15 cm polypropylene meshes were used, secured with either continuous or interrupted sutures. The materials used and the implantation technique were in accordance with clinical standards.

If complications occurred, they were recorded, including their nature, date of occurrence, and treatment measures undertaken. Hematomas were evacuated, and if signs of infection occurred, antibacterial therapy was prescribed. All cases of complications were recorded in a standardized form created specifically for this study. The surgeries were performed by the same surgical team with comparable levels of experience in both surgical techniques. The study protocol was reviewed and approved by the institutional ethics committee of the hospital where the study was conducted. The study was performed in accordance with the principles of the Declaration of Helsinki. All procedures were performed by surgeons from the same department with more than 10 years of clinical experience. In cases where a resident participated, the procedure was conducted under the direct supervision of an experienced specialist.

## 3. Results

The primary objective of the analysis was to assess differences between the two most commonly used techniques: laparoscopic TAPP and traditional open Lichtenstein repair. The baseline characteristics and main outcomes of the study groups are presented in Table 1.

Participants ranged in age from 19 to 92 years. The TAPP group was predominantly male, accounting for 96.7% of the total number of patients (29 of 30). Women were minimally represented in this subgroup, accounting for only one case (3.3%). The mean age of patients in this group was 55.6 years. The vast majority of these patients were of working age.

The Lichtenstein group consisted of 142 patients, with a male predominance, but the level of gender imbalance was less pronounced compared to the TAPP group. Women comprised 11.3% (16 of 142), and men 88.7%. The mean age of patients in this group was higher—64.4 years. Furthermore, most patients suffered from comorbidities, primarily cardiovascular and endocrine ones.

It was also noted that in the age group over 70, the open approach was used in the vast majority of cases. Of the 47 patients classified in this category, only 2 underwent TAPP surgery, representing less than 5%.

Of the 172 cases, 30 procedures were performed using the TAPP technique, all classified as elective. This is due to the need for initial patient preparation, comprehensive diagnostics, and the organizational specifics of laparoscopic procedures, which require the participation of trained personnel and the availability of appropriate equipment.

In the Lichtenstein group, 12 of the 142 procedures (8.5%) were performed urgently due to an incarcerated hernia, suspected ischemia of the hernia contents, or pain requiring immediate intervention. The remaining 130 procedures were elective. This reflects typical clinical practice, where an open approach is used for all types of hernias, including complex ones.

Analysis of the length of hospital stay after surgery revealed statistically significant differences between the study groups (*p* < 0.05). In the TAPP subgroup, 86.7% of the patients (26 of 30) were discharged on the third day after surgery and the remaining 13.3% (4 patients) on the fourth day. None of the patients in this subgroup remained in the hospital longer than four days. The mean length of stay was 3.13 days.

In the Liechtenstein group, significant variation was observed. Only 45.8% of the patients (65 of 142) were discharged on the third day. In 54.2% of the patients, the hospitalization duration was between 4 and 7 days, with a mean of 4.76 days. Patients undergoing emergency surgery had a longer average hospital stay of 5.4 days (*p* < 0.05). In patients undergoing elective Liechtenstein surgery, this period was 4.5 days. The TAPP method demonstrated an advantage in shortening hospital stay, which may be important in conditions of limited hospital beds and when organizing medical care in hospitals.

A more detailed analysis of hospitalization periods revealed that the length of hospital stay depended not only on the intervention method but also on concomitant clinical factors. For example, in patients from the Liechtenstein group who were hospitalized for more than 5 days, 82% of patients had comorbidities—hypertension, coronary artery disease, and type 2 diabetes. This prolonged the perioperative period due to the need to adjust pharmacological therapy and monitor the patient’s condition.

In the Liechtenstein surgical group, only two of the 12 emergency surgeries resulted in discharge on the third day. The remaining patients stayed in the hospital for 5 to 7 days, with an average of 5.4 days. This highlights the fact that the emergency nature of the intervention directly affects the length of hospital stay, which in turn increases the workload of surgical departments and the treatment resource intensity.

Early postoperative complications considered in the study included hematomas, seromas, wound infection, bleeding, acute urinary retention, and cases requiring reoperation. All complications were recorded within the first three days after surgery.

No early postoperative complications were observed in the TAPP subgroup during the in-hospital observation period. No patients complained of pain, swelling, wound discharge, or other signs of inflammation. There were no cases of rehospitalization or prolonged hospital stay for medical reasons.

In the Lichtenstein group, complications were reported in 6.4% of patients (9 of 142). The most common type of complication was soft tissue hematomas in the groin area—5 cases, 3 of which required evacuation under local anesthesia. In two cases, the hematomas were accompanied by a rise in body temperature and were considered infected. In these cases, antibacterial therapy with dynamic monitoring was administered.

Wound infection was diagnosed 2–3 days after the procedure, accompanied by an increase in body temperature up to 38.5 °C, local hyperemia, and purulent discharge. Two patients received local wound care, and in one case surgical correction was necessary. Negative dynamics were observed in only one patient, who underwent emergency intervention.

Implant removal was the most serious complication in this group. In a hospitalized patient the wound dehisced and purulent discharge appeared on the third day post-surgery. Bacteriological tests revealed the presence of multiresistant flora, which required not only mesh removal but also long-term antibacterial therapy. The patient remained in the hospital for 11 days and was readmitted three weeks later due to hernia recurrence.

The majority of the patients were classified as ASA classes I–II (61.1% in the TAPP group and 56.4% in the Lichtenstein group). Patients undergoing open surgery had a higher incidence of significant comorbidities, as reflected by a higher percentage of ASA classes III–IV (43.6% vs. 16.7%, *p* = 0.01, chi-square test). Comorbidities were more frequently observed in the Lichtenstein group, particularly cardiovascular diseases, including hypertension and coronary artery disease, as well as metabolic conditions such as type 2 diabetes. These conditions were less common in the TAPP group.

## 4. Discussion

The data obtained during the study confirmed the differences between the TAPP and Lichtenstein methods in terms of the incidence of early postoperative complications and hospitalization duration. These results are consistent with several previous publications but also point to important regional characteristics specific to Polish clinical practice.

Comparison with other studies shows that the TAPP method is associated with less trauma and a more favorable postoperative course. A previous study noted a lower risk of infection with the laparoscopic approach, as well as a reduction in pain intensity in the first few days after surgery [7]. Similarly, another study indicated TAPP as the preferred method when appropriate equipment and a trained surgeon are available [11]. Furthermore, according to another study, short-term quality of life after robotic-assisted extraperitoneal hernia repair was significantly higher compared to open and laparoscopic methods, and patients returned to daily activities more quickly, despite similar results in most other parameters [13].

It should be noted, however, that the TAPP method was primarily used in middle-aged patients, while the Lichtenstein method was more frequently used in older patients. This tendency may be explained by several factors, including the need for general anesthesia in laparoscopic procedures, patient positioning, pneumoperitoneum, and greater caution when considering laparoscopy in elderly patients. In the present study, open procedures were primarily performed under spinal anesthesia, which is often preferred in elderly patients with higher anesthetic risk. This may have contributed to the higher proportion of older patients in the Lichtenstein group and should be considered a potential source of selection bias when interpreting the results. These findings are partially consistent with previous reports, indicating that elderly patients with complex inguinal hernias and significant comorbidities are more likely to undergo open or urgent procedures, which are associated with an increased risk of postoperative complications [9].

The general trend towards a lower complication rate with the laparoscopic approach is confirmed by both this study and the work of other authors. It should be noted that in most cases, laparoscopy is used in patients who have a higher likelihood of a positive outcome, which may further distort the perception of its safety and effectiveness. Patients selected for laparoscopic surgery are typically in a more stable physical condition, have fewer comorbidities, and undergo thorough preoperative preparation. However, open surgery is often performed in emergency situations or in patients with high surgical risk, which naturally increases the likelihood of adverse effects [14,15,16]. Therefore, according to the international GLACIER study, which included 1014 surgeons from 81 countries, laparoscopic methods (specifically TAPP) proved to be more beneficial for bilateral and recurrent inguinal hernias, while the Lichtenstein technique remains the most commonly used technique for open hernioplasty. Chronic pain was also observed to be more common after open surgery (5%) compared with minimally invasive surgery (1%) [17]. Therefore, differences in outcomes between methods may be due not only to the surgical technique itself but also to selection factors, which should be considered when interpreting results and comparing data from different authors. This highlights the need for standardized risk assessment scales and multivariate analysis to obtain a more objective picture of the effectiveness of each method in clinical practice.

One key factor influencing the study results was the urgency of the intervention. In the Liechtenstein group, emergency procedures accounted for 8.5% of cases, while in the TAPP group, none were performed. A previous study emphasizes that the emergency nature of the procedure is associated with an increased incidence of early complications, which is explained by the lack of appropriate preoperative preparation and the more severe condition of the patients [12]. The data obtained fully confirm this: among patients undergoing emergency interventions using the Liechtenstein method, the incidence of complications was significantly higher compared to elective cases. This may be due not only to the urgency of the situation but also to the inability to conduct timely preoperative testing, adjust concomitant therapy, and provide adequate pain relief. Furthermore, in the case of emergency hospitalization, there is often no choice of treatment method, which limits the use of laparoscopic techniques that require specialized equipment and trained personnel. In such circumstances, the more accessible and technically simpler open method is preferred, despite the potentially higher risk of complications. Urgency of intervention is therefore not only a clinical indicator of disease severity but also a systemic factor influencing the pattern of complications and overall treatment outcomes. However, experienced surgeons consider laparoscopic techniques (TAPP and TEP) to be preferable because they are associated with faster recovery and lower levels of chronic pain compared to open techniques [18].

This study did not distinguish between primary and secondary hernias, nor did it consider other surgical methods that may play a significant role in subsequent treatment. Therefore, a previous study demonstrated that surgical procedures for repairing recurrent inguinal hernias are more complex and require more time and resources than primary interventions, especially after Shouldice and open mesh repairs [19].

In the present study, the mean age of patients with complications was 71.3 years, which may indicate a higher likelihood of complications in older adults. This is consistent with the results of a previous study, which identified age and comorbidities as the main predictors of poor outcome. In this study, complications in the Lichtenstein group were virtually nonexistent in patients under 60 years of age, whereas their incidence exceeded 10% in patients over 70 years of age. These findings suggest that patient age may be associated with an increased risk of postoperative complications, likely reflecting a higher prevalence of comorbidities in older patients, while local complications such as hematomas are more likely related to surgical technique [8,20].

However, the study did not take into account the financial aspects of hernia treatment. According to a study conducted in Germany, age and the presence of comorbidities have a significant impact on treatment costs [21]. At the same time, an analysis showed that inguinal hernia surgery improves quality of life in both older and younger patients, particularly in terms of physical function and pain reduction, and the complication rate is not age-dependent [22]. According to a recent study, the TAPP method for unilateral inguinal hernias provides better postoperative pain and quality of life scores in the early period (3 and 14 days) compared with open Bassini repair, with these differences leveling off after 2 and 6 months [23]. Although age is associated with an increased risk of complications, surgery has a positive impact on the quality of life in patients regardless of age.

Limitations of this study include its retrospective nature and the relatively small TAPP sample size. It should also be noted that the study was conducted in a district-level hospital, which reflects real-world surgical practice outside high-volume specialized centers. This may explain the relatively low number of laparoscopic procedures and should be taken into account when interpreting the results. It should also be noted that the length of hospital stay in our study was influenced by institutional and healthcare system-related organizational factors, including local hospitalization protocols. This may limit direct comparison with centers utilizing day-surgery pathways. A further limitation of this study is the lack of long-term follow-up, which precludes assessment of delayed complications such as chronic postoperative pain or entrapment neuropathy. Future promising directions include expanding the sample size, incorporating multicenter data, and using multivariate analysis to consider the influence of several variables simultaneously.

Despite these limitations, the results of this analysis can serve as a basis for formulating recommendations for optimizing the approach to selecting a surgical method for inguinal hernia repair based on patient age and clinical circumstances. These findings confirm the need for more active implementation of laparoscopic technologies, especially in elective surgery, and emphasize the importance of age-based risk stratification when using open techniques.

Therefore, a discussion of the obtained results in the context of contemporary scientific literature and the characteristics of the national healthcare system allows for drawing rational conclusions and formulating practical recommendations, which will be presented in the conclusion.

## 5. Conclusions

A comparative analysis of the TAPP and Lichtenstein inguinal hernia repair methods identified a number of significant differences that are clinically and organizationally important. Based on a retrospective study of 172 cases, the laparoscopic TAPP technique provided favorable short-term outcomes compared to the open method, particularly in terms of early postoperative complications and length of hospital stay.

Patients undergoing the TAPP procedure experienced no early complications during the in-hospital observation period, suggesting favorable short-term outcomes with this technique. Furthermore, their average hospital stay was significantly shorter compared to patients undergoing Lichtenstein surgery. It is important to note that the TAPP method was primarily used in working-age patients, which may also have contributed to the favorable outcomes.

In the Lichtenstein group, complications were reported in 6.4% of patients, with the incidence increasing proportionally with age. The highest percentage of adverse events occurred in patients over 70 years of age. It was also determined that emergency procedures are associated with an increased incidence of complications and longer hospital stays.

The results of this study suggest that the TAPP approach may be associated with favorable short-term outcomes in selected patients undergoing elective procedures, while the Lichtenstein method remains an important option in elderly patients, those with contraindications to general anesthesia, and in resource-limited settings.

The choice of surgical technique should be individualized based on patient characteristics and clinical context. Further prospective multicenter studies are required to confirm these findings.

## Figures and Tables

**Table 1:** Frequency of complications and duration of hospitalization depending on the surgical method.

Indicator	TAPP (*n* = 30)	Liechtenstein (*n* = 142)
Mean age (years)	55.6	64.4
Men, %	96.7	88.7
Elective operations, %	100	91.5
Emergency operations, %	0	8.5
Mean length of hospital stay (days)	3.13	4.76
Complication rate, %	0	6.4
Hematomas, n	0	5
Infected wounds, n	0	3
Repeated interventions, n	0	1

## Data Availability

The data presented in this study are not publicly available due to privacy and ethical restrictions related to patient confidentiality. However, anonymized data may be made available from the corresponding author upon reasonable request and with permission of the Ethics Committee.
